# A Cannabigerol Derivative Suppresses Immune Responses and Protects Mice from Experimental Autoimmune Encephalomyelitis

**DOI:** 10.1371/journal.pone.0094733

**Published:** 2014-04-11

**Authors:** Francisco J. Carrillo-Salinas, Carmen Navarrete, Miriam Mecha, Ana Feliú, Juan A. Collado, Irene Cantarero, María L. Bellido, Eduardo Muñoz, Carmen Guaza

**Affiliations:** 1 Neuroimmunology Group, Functional and System Neurobiology Department, Instituto Cajal, Madrid, Spain; 2 VivaCell Biotechnology España, Parque Científico Tecnológico de Córdoba, Córdoba, Spain; 3 Instituto Maimónides de Investigación Biomédica de Córdoba (IMIBIC), Hospital Universitario Reina Sofía, Universidad de Córdoba, Córdoba, Spain; Iibb-Csic, Idibaps, Ciberned, Spain

## Abstract

Phytocannabinoids that do not produce psychotropic effects are considered of special interest as novel therapeutic agents in CNS diseases. A cannabigerol quinone, the compound VCE-003, has been shown to alleviate symptoms in a viral model of multiple sclerosis (MS). Hence, we studied T cells and macrophages as targets for VCE-003 and its efficacy in an autoimmune model of MS. Proliferation, cell cycle, expression of activation markers was assessed by FACs in human primary T cells, and cytokine and chemokine production was evaluated. Transcription was studied in Jurkat cells and RAW264.7 cells were used to study the effects of VCE-003 on IL-17-induced macrophage polarization to a M1 phenotype. Experimental autoimmune encephalomyelitis (EAE) was induced by myelin oligodendrocyte glycoprotein (MOG_35–55_) immunization and spinal cord pathology was assessed by immunohistochemistry. Neurological impairment was evaluated using disease scores. We show here that VCE-003 inhibits CD3/CD28-induced proliferation, cell cycle progression and the expression of the IL-2Rα and ICAM-1 activation markers in human primary T cells. VCE-003 inhibits the secretion of Th1/Th17 cytokines and chemokines in primary murine T cells, and it reduces the transcriptional activity of the IL-2, IL-17 and TNFα promoters induced by CD3/CD28. In addition, VCE-003 and JWH-133, a selective CB2 agonist, dampened the IL-17-induced polarization of macrophages to a pro-inflammatory M1 profile. VCE-003 also prevented LPS-induced iNOS expression in microglia. VCE-003 ameliorates the neurological defects and the severity of MOG-induced EAE in mice through CB2 and PPARγ receptor activation. A reduction in cell infiltrates, mainly CD4^+^ T cells, was observed, and Th1 and Th17 responses were inhibited in the spinal cord of VCE-003-treated mice, accompanied by weaker microglial activation, structural preservation of myelin sheets and reduced axonal damage. This study highlights the therapeutic potential of VCE-003 as an agent for the treatment of human immune diseases with both inflammatory and autoimmune components.

## Introduction

Multiple sclerosis (MS) is a chronic inflammatory demyelinating disease of the CNS and the main cause of non-traumatic neurological disability in young adults. Supported by experimental evidence largely collected from an important animal model, experimental autoimmune encephalomyelitis (EAE), MS is considered a predominantly T cell-mediated autoimmune disease. Although the exact cause of MS remains unclear, CNS inflammation is a key element in the pathophysiology of this disease. The histopathological hallmarks of MS include neuroinflammation, reflected by the migration of leukocyte infiltrates into the central nervous system (CNS), and the loss of myelin and axonal damage [1]. Indeed, Focal plaques of demyelination are observed in the white matter of the brain and spinal cord [2]. EAE is a CD4^+^ T cell–mediated autoimmune disease characterized by perivascular CD4^+^ T cell and mononuclear cell inflammation, with subsequent primary demyelination of axonal tracks in the CNS that provokes progressive hind-limb paralysis [3]. In the EAE model, nervous tissue inflammation is characterized by the activation of microglia [4], [5], which may amplify damage by releasing mediators of toxicity and cytokines. Based on the essential role of the autoimmune response in MS and in EAE [6], immunomodulatory agents have been tested and approved for MS therapy. However, these treatments are associated with side effects and their effectiveness is limited.

Phytocannabinoids (pCBs) that do not produce psychotropic effects, such as cannabidiol (CBD), cannabigerol (CBG), Δ9 tetrahydrocannabivarin (Δ9THCV) and cannabidivarin (CBDV) [7], are considered of special interest as novel therapeutic agents in CNS diseases. In the CNS, there is evidence that CB1 receptors play a fundamental role in neuroprotection [8], while CB2 receptors expressed primarily in microglia regulate microglial neurotoxicity [9]. Besides the classical membrane CB receptors, nuclear PPARγ receptors have also been shown to be activated by pCBs, and since they attenuate neuroinflammation, pCBs may be of therapeutic interest for the treatment of inflammatory diseases [10].

Preclinical and clinical data have shown that both Th1 and Th17 cells are associated with autoimmune diseases, including MS [11]. Moreover, there is evidence that Th17 cells play a crucial role in EAE, perhaps more intensely in the initial phases of disease, and Th1 cells probably contribute to disease pathogenesis by exerting a more pronounced effect later in its development. EAE is strongly suppressed in mice lacking IL-17 or the IL-17R, and specific inhibition of IL-17 attenuates CNS inflammation [12]–[14]. However, the mechanism by which IL-17 participates in the pathogenesis of EAE is still unclear. It was recently shown that IL-17 mediates inhibition of oligodendrocyte maturation and deletion of the NF-κB activator 1 in NG2^+^ glial cells, a key transducer of IL-17 signaling, reduces EAE severity [15]. IL-17 also modulates the differentiation of macrophages to the pro-inflammatory M1 phenotype [16]. Moreover, it has been suggested that IL-17 could disrupt tight junctions in the blood-brain barrier (BBB) through the induction of reactive oxygen species (ROS), allowing peripheral autoimmune cells to penetrate the CNS [17], [18]. Therefore, inhibition of IL-17 production by Th17 cells, and IL-17 signaling on target cells, has opened new avenues to find an effective therapy for MS.

Although there are few studies about the pharmacological actions of CBG, one of the most significant in the context of inflammation is its benefits in experimental inflammatory bowel [19] by reducing nitric oxide production by macrophages through activation of CB2 receptors. We have developed a series of new cannabinoid quinones, including the CBG quinone named VCE-003 that is a PPARγ and CB2 receptor agonist [20]. We have shown that VCE-003 alleviates neuroinflammation and motor deficits in the viral TMEV-IDD model of MS [20], however, its mode of action and cellular targets are still to be defined. In the present study, we investigated whether T lymphocytes might be direct targets of VCE-003 by using *in vitro* approaches. In human primary T cells, VCE-003 inhibited CD3/CD28-induced proliferation, cell cycle progression and the expression of the activation markers IL-2Rα and ICAM-1. VCE-003 also inhibited the secretion of Th1/Th17 cytokines and chemokines in primary T cells, and it reduced the transcriptional activity of the IL-2, IL-17 and TNFα promoters induced by CD3/CD28 in Jurkat cells. In addition, VCE-003 blunted IL-17-induced polarization of macrophages to a pro-inflammatory M1 profile. Therefore, we assessed whether VCE-003 may be effective in an autoimmune model of MS, such as EAE. We immunized C57BL/6 mice with MOG_35–55_ peptide to induce EAE, and tested the effect of treatment with VCE-003 on the development and progression of EAE. Our findings indicate that VCE-003 attenuated EAE by activating CB2 and PPARγ receptors. Specifically, we observed a reduction of cell infiltrates, mainly that of CD4^+^ cells, and inhibition of Th1 and Th17 responses in the spinal cord of VCE-003-treated mice, accompanied by decreased microglial activation, structural preservation of myelin sheets and reduced axonal damage.

## Methods

### Cell lines and reagents

Jurkat, BV2 and RAW264.7 cells were maintained at 37°C in a humidified atmosphere containing 5% CO_2_ in RPMI supplemented with 10% fetal calf serum (FCS), 2 mM L-glutamine and 1% (v/v) penicillin/streptomycin (complete medium). VCE-003 was synthesized as previously described [20], while AM630, T0070907 and WIN-55,212-2 were obtained from Cayman Chem (Ann Arbor, MI, USA), and JWH-133 was purchased from Tocris Bioscience (Bristol, UK). All other reagents were from Sigma Co (St Louis, MO, USA).

### Induction and assessment of EAE

All experiments were performed in strict accordance with EU and governmental regulations (Decret 53/2013 BOE n°34 and Comunidad de Madrid: ES 280790000184). The Ethics Committee on Animal Experimentation of the Instituto Cajal, CSIC approved all procedures described in this study (protocol number: 2013/03 CEEA-IC). Measures to improve welfare assistance and clinical status as well as endpoint criteria were established to minimise suffering and ensure animal welfare. Briefly, wet food pellets are placed on the bed-cage when the animals begin to develop clinical signs to facilitate access to food and hydration. Mice suffering severe disease (score 5) were euthanized in accordance with our ethical guidelines. Female C57BL/6 mice were purchased from Harlan (Barcelona, Spain) and housed in our animal facilities (Instituto Cajal, CSIC, Madrid, Spain) under the following controlled conditions: 12 h light/dark cycle; temperature 20°C (±2°C) and 40–50% relative humidity with free access to standard food and water. EAE was induced in female mice at 6–8 weeks of age by subcutaneous immunization with MOG_35–55_ (300 µg: peptide synthesis section, CBM, CSIC, Madrid, Spain) and 200 µg of *Mycobacterium tuberculosis* (H37Ra Difco, Franklin Lakes, NJ, USA) in a 1∶1 mix with incomplete Freund's adjuvant (CFA, Sigma-Aldrich, Madrid, Spain). On the same day and 2 days later, mice were injected intraperitoneally (ip) with 200 ng of pertussis toxin (Sigma-Aldrich, Madrid, Spain) in 0.1 ml PBS. Control animals (CFA) were inoculated with the same emulsion without MOG and they did not receive pertussis toxin. Treatment started at day 6 post-immunization (p.i.) and consisted in daily injections of VCE-003 (5 mg/kg) or of the vehicle alone (DMSO/PBS) for the following 21 days. In some experiments, mice received a combination of VCE-003 and the CB2 antagonist AM630 (2 mg/kg), administered 15 min before VCE-003, or the combination of VCE-003 and the PPARγ antagonist T0070907 (5 mg/kg) injected 15 minutes before VCE-003. The mice were examined daily for clinical signs of EAE and disease scores were measured as follows: 0, no disease; 1, limp tail; 2, limp tail and hind limb weakness; 3, hind limb paralysis; 4, hind limb and front limb paralysis; 5, moribund and death. Groups: Intact, CFA, EAE + VEH; EAE + VCE-003; EAE + VCE-003 + AM630*; EAE + VCE-003 + T0070907. All animals were sacrificed 28 days (p.i.) for further analysis. * 4 animals from the EAE + VCE-003 + AM630 group were sacrificed due to disease severity (score 5).

### Tissue processing and Immunohistochemistry (IHC)

Mice were anesthetized by ip administration of pentobarbital (50 mg/kg body weight) and they were transcardially perfused with saline. Spinal cords were fixed in 4% paraformaldehyde in 0.1 M PBS, washed in 0.1 M PBS, cryoprotected with a 15% and then a 30% solution of sucrose in 0.1 M PBS, and frozen at −80°C. Free-floating thoracic spinal cord sections (15/30 µm thick) were washed three times for 10 min with 0.1 M phosphate buffer (PB). Endogenous peroxidase activity was inhibited with 50% methanol and 1.66% hydrogen peroxide. The sections were blocked with 0.1% Triton X-100 and 5% animal serum and then incubated overnight at 4°C in blocking buffer with the primary antibody. For IHC in 30 µm sections, microglia cells were stained with a rabbit anti-mouse Iba-1 antibody (1∶1,000; Wako Chemical Pure Industry, Osaka, Japan) and a primary rat anti-mouse CD4 antibody (1∶1,000; BD Pharmingen; San Diego, CA, USA) was used to detect CD4+ T cells (sections of 30 µm thick). In 15 µm thick sections, a mouse anti-mouse SMI32 antibody (1∶1,000; Covance; San Diego, CA, USA) was used to detect axonal damage, while for immunofluorescence, myelin was stained with a RIP antibody (1∶1,000; DSHB; University of Iowa, IA, USA) and axons with a Neurofilament H antibody (1∶1,000; Millipore; Billerica, MA, USA). After incubation with the primary antibody the sections were rinsed with PB three times for 10 min and then incubated for 1 h with the secondary antibody: biotinylated goat anti-rabbit (Iba-1); fluorescent goat anti-rabbit (Neurofilament H); biotinylated rabbit anti-rat (CD4); fluorescent goat anti-mouse (RIP); and biotinylated horse anti-mouse (SMI32). For immunofluorescence visualization, sections were rinsed with PB three times for 10 min and mounted. For immunostaining with DAB, after rinsing the sections were incubated for 1 h with a biotin-peroxidase complex (Vector Laboratories Inc.; Burlingame, CA, USA) and then with the chromogen 3,3′diaminobenzidine tetrahydrochloride (DAB; Sigma-Aldrich; St. Louis, MO, USA). After staining, the sections were dehydrated, cleared with xylene and coverslipped. In all cases specificity of staining was confirmed by omitting the primary antibody. Staining was quantified using the Image J software designed by the National Institutes of Health (NIH; Bethesda, MD, USA).

### Luxol Fast Blue (LFB) staining

Free-floating thoracic spinal cord sections (15 µm thick) were washed three times for 10 min in 0.1 M PB. The samples were dehydrated in increasing concentrations of ethanol, from 70% to 95% and they were then incubated in LFB solution overnight at 56°C. The following day, the excess stain was rinsed off with 95% ethyl alcohol, and the slides differentiated in the lithium carbonate solution for 30 seconds. Sections were dehydrated, cleared with xylene and cover slipped.

### Inflammatory infiltrates

Thoracic slices were stained with Nissl solution at 50°C for 10 min and the sections were then dehydrated in 100% ethyl alcohol, cleared in xylene and cover slipped. Inflammatory infiltrates were evaluated on a scale of 0 to 4, the score reflecting the amount of infiltrates in the thoracic spinal cord sections. A score of 4 reflects the largest number of infiltrates with all the intermediate scores (1, 2 and 3) to define the increase in the density of infiltrates in the spinal cord tissue.

### Microscopy and image analysis

Six thoracic spinal cord sections per animal from at least 6 animals per group were taken. Staining was quantified using the Image J software (NIH; Bethesda, MD, USA). Sections were analyzed by immunofluorescence on a Leica TCS SP5 confocal microscope and with a Zeiss Axiocam high-resolution digital color camera for IHC.

### Real-time RT-PCR

Total RNA was isolated from the spinal cord of control (CFA), EAE + VEH and EAE + VCE-003 treated animals. Spinal cord sections from at least 6 animals were analyzed per group. Total RNA (1 µg) was retrotranscribed using the iScript™ cDNA Synthesis Kit (Bio-Rad; Hercules, CA, USA), and the cDNA generated was analyzed by real-time PCR, using the iQ™ SYBR Green Supermix (Bio-Rad; Hercules, CA, USA). Real-time PCR was performed using a CFX96 Real-Time PCR Detection System (Bio-Rad; Hercules, CA, USA). The GAPDH gene was used to standardize mRNA expression in each sample and gene expression was quantified using the 2-ΔΔCt method. The oligonucleotide primer sequences used are given in Table 1.

**Table 1:** The mouse primer sequences used in quantitative Polymerase Chain Reactions.

Genes	Forward	Reverse
IL-6	5′ -GAACAACGATGATGCACTTGC- 3′	5′ -TCCAGGTAGCTATGGTACTCC- 3′
IL-1β	5′ -CTCCACCTCAATGGACAGAA- 3′	5′ -GCCGTCTTTCATTACACAGG- 3′
Ccl2	5′ -GGGCCTGCTGTTCACAGTT- 3′	5′ -CCAGCCTACTCATTGGGAT- 3′
Ccl4	5′ -AACAACATGAAGCTCTGCGT- 3′	5′ -AGAAACAGCAGGAAGTGGGA- 3′
IFNγ	5′-CTCAAGTGGCATAGATGTGGAAG-3′	5′-GCTGGACCTGTGGGTTGTTGA-3′
IL-17	5′-CCTCAGACTACCTCAACCGTTC-3′	5′-TTCATGTGGTGGTCCAGCTTTC-3′
iNOS	5′ -AACGGAGAACGTTGGATTTG-3′	5′-CAGCACAAGGGGTTTTCTTC-3′
ICAM-1	5′ -CAGATGCCGACCCAGGAGAG-3′	5′ -ACAGACTTCACCACCCCGATG-3′
TNFα	5′ -AGAGGCACTCCCCCAAAAGA-3′	5′ -CGATCACCCCGAAGTTCCCATT-3′
GAPDH	5′-TGGCAAAGTGGAGATTGTTGCC-3′	5′-AAGATGGTGATGGGCTTCCCG-3′
18S	5′- ATGCTCTTAGCTGAGTGTCCCG-3′	5′ -ATTCCTAGCTGCGGTATCCAGG-3′

### Western blotting

After treatments, BV2 cell cultures were lysed in Tris-buffered saline (TBS, pH 7.6) containing 10% glycerol, 1% Nonidet P-40, EDTA 1 mM, EGTA 1 mM plus a complete protease inhibitors cocktail (Roche Diagnostics; Mannheim, Germany). The cell lysates were mixed with 5x Laemmli sample buffer and boiled for 5 min. Equal amounts of protein (30 µg) were resolved on 10% SDS-PAGE and electroblotted for 70 min at 90 V and 4°C to nitrocellulose (Amersham Biosciences; Amersham, UK). The membranes were blocked for 1 h at RT in 5% (w/v) dry skim milk (Sveltesse, Nestlé; Barcelona, Spain) diluted in TBS with 0.1% Tween® 20 (TBST) and the membranes were then probed overnight at 4°C with the antibody against iNOS diluted in 5% milk-TBST (1∶1,000; BD Pharmingen; San Diego, CA, USA). After extensive washing with 5% milk-TBST and antibody binding was detected for 1 h at room temperature with a horseradish peroxidase-conjugated anti-goat secondary antibody (1∶4,000; Bio-Rad; Hercules, CA, USA), which was visualized after rinsing enhanced chemiluminescence (Amersham Biosciences; Amersham, UK). The blots were stripped in 62.5 mM Tris-HCl, pH 6.8, containing 2% SDS and 0.7% β-mercaptoethanol and the were reprobed with a monoclonal antibody against α-Tubulin (1∶10,000; Sigma; Madrid, Spain).

### Isolation of human peripheral T cells and proliferation assays

All experiments conducted on human material were approved by the Ethics committee for Biomedical Research of the University of Córdoba. Peripheral blood mononuclear cells (PBMC) were isolated by Ficoll–Hypaque density gradient centrifugation from buffy coats of blood donors obtained from the blood bank of the Hospital Universitario Reina Sofía Córdoba, Spain. Human peripheral T cells were isolated using a Pan T cell isolation kit (Miltenyi Biotech; Madrid, Spain) and 10^5^ cells were cultured in triplicate in 96-well round bottom microtiter plates in 200 µl of complete medium. The cells were then stimulated with the anti-CD3 antibody OKT3 (coated 1 µg/ml) and the anti-CD28 antibody 15E8 (0.5 µg/ml) in the presence or absence of increasing concentrations of VCE-003. The cultures were maintained for three days and pulsed with 0.5 µCi [^3^H]TdR/well for the last 12 h of culture, measuring the radioactivity incorporated into DNA by liquid scintillation counting.

### Cytofluorimetric analyses of cell surface antigen, cell cycle and cellular division

To analyze the cell cycle and measure CD25 expression, peripheral T cells (10^6^/ml) were stimulated with for 72 h anti-CD3 and anti-CD28 antibodies in 24 well plates in a total volume of 2 ml complete medium in the presence or absence of different concentrations of VCE-003. The expression of CD25 and CD54 at the cell surface was measured by fluorescence using a specific mAb (CD54: PE anti-human CD54; BD Pharmingen; San Diego, CA, USA; CD25: Mouse Anti-Human interleukin-2 receptor; Dako; Glostrup, Denmark), which was analyzed by flow cytometry in a FACSCAnto II flow cytometer. To analyze the DNA profile, the cells were washed in PBS and fixed in ethanol (70%, for 24 h at 4°C), before digesting their RNA (RNAseA, 50 U/ml) and staining them with propidium iodide (20 µg/ml) to be analyzed by cytofluorimetry. Ten thousand gated events were collected per sample and the percentage of cells in each phase of the cell cycle was determined. To analyze cell division, purified T cells were stained with carboxy-fluorescein succinimidyl ester (CSFE) and stimulated with anti-CD3 (coated 1 µg/ml) and anti-CD28 (coated 0.5 µg/ml) antibodies in the presence or absence of VCE-003 for 6 days and the percentage of proliferating cells (defined as weak CFSE fluorescence) was determined by flow cytometry.

### Cytokine detection

Peripheral T cells (10^6^/ml) were stimulated with anti-CD3 (coated 1 µg/ml) and anti-CD28 (0.5 µg/ml) antibodies in 24 well plates for 72 h in the presence or absence of different concentrations of VCE-003. The supernatants were collected and the cytokines and chemokines were detected using the semiquantitative Human Cytokine Array Kit, Panel A (R&D System; Minneapolis, MN, USA) according to the manufacturer's recommendations. Pixel densities on developed X-Ray films were collected with a scanner and analyzed using the ImageJ processing and analysis program (NIH; Bethesda, MD, USA).

### Transient transfection and Luciferase assays

Jurkat cells (10^7^/ml) were transiently transfected with the IL-2-Luc, TNF-Luc and IL-17-Luc plasmids using the Lipofectine™ reagent (Life Technologies; Madrid, Spain), according to the manufacturer's recommendations. Twenty-four hours after transfection, the cells were pre-treated with VCE-003 for 30 min and stimulated for 6 h with the OKT3 (coated 1 µg/ml) and the anti-CD28 antibody 15E8 (0.5 µg/ml). The cells were then lysed in 25 mM Tris-phosphate buffer [pH 7.8] containing 8 mM MgCl_2_, 1 mM DTT, 1% Triton X-100, and 7% glycerol. The luciferase activity was quantified on an Autolumat LB 953 (EG&G Berthold; Oak Ridge, TN, USA), following the manufacturer's instructions (Luciferase assay kit, Promega; Madison, WI, USA), and the protein concentration was measured by the Bradford method. The pGL4.74 vector (Promega; Madison, WI, USA) that contains a constitutively expressed firefly luciferase gene served as an internal control to normalize transfection efficiency and firefly and renilla luciferase activities were measured using the Dual-Luciferase® reporter assay system (Promega; Madison, WI, USA). The background obtained with the lysis buffer was subtracted from each experimental value and the specific transactivation was expressed as the fold induction relative to the untreated cells. All the experiments were repeated at least three times.

### Pro-inflammatory cytokine production by IL-17-stimulated macrophages

Serum-starved RAW264.7 macrophages were pre-incubated with JWH-133 (Tocris Bioscience; Bristol, UK) or VCE-003 for 18 h, before they were exposed for an additional 24 hours to recombinant mouse IL-17 (50 ng/mL; R&D Systems; Minneapolis, MN, USA). The cells were collected in PBS and total RNA was extracted using the RNeasy Mini Kit (Qiagen; Hilden, Germany).

### CB2 functional assay

CHO-CB2 cells were transfected with the CRE-luc plasmid that contains six consensus cAMP responsive elements (CRE) linked to firefly luciferase. The cells were treated for 6 h with forskolin (FSK, 1 µM) 24 hours after transfection, in the presence or absence of either WIN-55,212-2 (1 or 10 µM) or VCE-003 (5 µM), and the luciferase activity measured in the cell lysates.

### Statistical analysis

For the course of EAE, the results are the mean ± SD from 6 to 11 mice per group. Statistical analysis was performed using the non-parametric Kruskal-Wallis test to compare the clinical score each day. For *in vitro* experiments, every assay was performed in duplicate and at least three independent experiments were performed. In each assay performed, the mean and SD or SEM were calculated, and plotted to visualize differences between the average values of the experimental populations. The sample population means were compared against the control population means using an unpaired two-tailed Student's t test. The p value obtained from the Student's t test analysis marks the probability of rejecting the null hypothesis, that is, that the events are independent, whereby: *p≤0.05, significant; **p≤0.01, very significant; and ***p≤0.005 or p≤0.001, highly significant.

## Results

### Immunosuppressive activity of VCE-003

**Figure 1:**
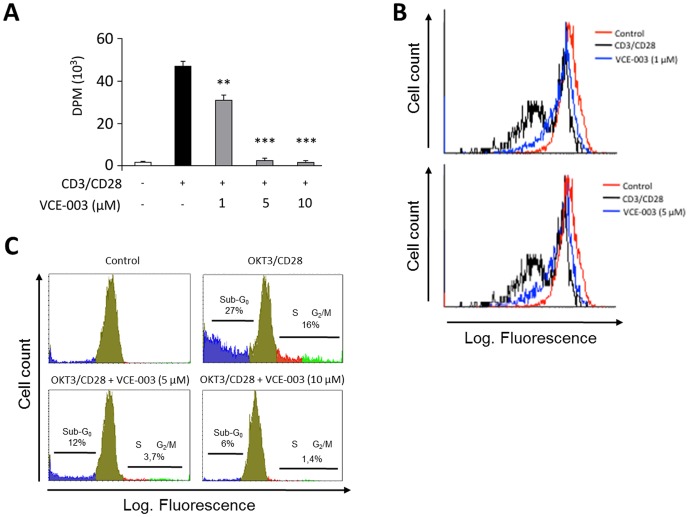
Effects of VCE-003 on T-cell proliferation. (A) Human peripheral T cells were stimulated with the OKT3 (1 µg/ml) and anti-CD28 (0.5 µg/ml) mAbs for 72 h in the presence or absence of increasing concentrations of VCE-003. Thymidine [^3^H] incorporation was measured by liquid scintillation and represented as the mean d.p.m ± SEM. Three independent experiments were performed. **p≤0.01 and ***p≤0.005 in an unpaired two-tailed Student's *t* test. (B) T cells were stained with CFSE, pre-treated with VCE-003 and stimulated with the CD3/CD28 mAbs for 6 days. Cell division was identified by flow cytometry. (C) T-cells were pre-treated with VCE-003 and stimulated with the CD3/CD28 mAbs for 72 h. The percentage of subdiploid cells (sub-G_0_), and cells entering the S and G_2_/M phases of the cell cycle are indicated. The results are representative of three independent experiments.

It is believed that myelin specific T cells of a Th1 and Th17 phenotype can mediate EAE [21]. Thus, to investigate the effect of VCE-003 on T cell activation we isolated human peripheral T cells that were stimulated by the mAb mitogens anti-CD3 and anti-CD28. DNA synthesis in CD3/CD28-stimulated T cells, measured by [^3^H]-TdR uptake, was markedly inhibited by VCE-003 in a concentration dependent manner (Figure 1A). This effect of VCE-003 on cell proliferation was confirmed by stimulating primary T cells with the CD3/CD28 mAbs for 6 days, and evaluating the proportion of CSFE-stained daughter cells by flow cytometry. We found that VCE-003 clearly prevented CD3/CD28-induction of T cell division even at the lowest concentration tested (Figure 1B).

T-cell receptor (TCR) engagement can induce different functional outcomes, such as activation, proliferation or apoptosis, and a second signal provided by CD28 can rescue T cells from TCR-induced apoptosis and promote proliferation [22]. Unstimulated T cells remained largely in the G_0_/G_1_ phase of the cell cycle (Figure 1C) whereas three days after activation with CD3/CD28, T cells were full cycling and progressed through the S, G_2_ and M phases of the cell cycle (16% of cells), while exposure to VCE-003 (5 µM) almost completely prevented the entry of the cells into the S-phase of the cell cycle (Figure 1C). Interestingly, VCE-003 also prevented CD3/CD28 from inducing apoptosis, evident through the percentage of hypodiploid cells (sub G_0_, 27%). These results indicate that at the doses used, VCE-003 did not induce cytotoxicity or apoptosis in primary T cells in culture.

**Figure 2:**
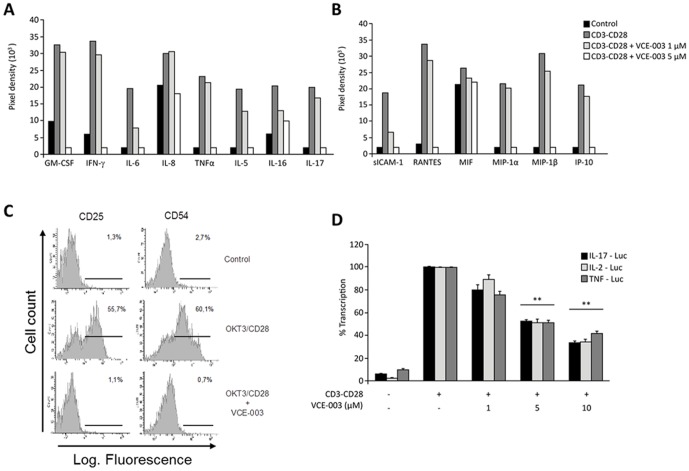
Effects of VCE-003 on T-cell activation. (A) Human peripheral T cells were stimulated for 72 h with the OKT3 (1 µg/ml) and anti-CD28 (0.5 µg/ml) mAbs in the presence or absence of increasing concentrations of VCE-003, and the culture supernatants were collected and assayed using a R&D Systems Human Cytokine Array system (A, cytokines; B, chemokines and sICAM-1). (C) Human peripheral T cells were stimulated as indicated in 2A for 72 h, and the expression of CD25 and CD54 was detected by flow cytometry. The numbers represent the percentage of CD25^+^ and CD54^+^ cells and they are representative of three different experiments. (D) Jurkat T cells transfected with TNFα, IL-17 or IL-2 promoter luciferase reporter plasmids were treated for 30 min with increasing concentrations of VCE-003, and then stimulated with OKT3 (1 µg/ml) and anti-CD28 (0.5 µg/ml) mAbs for 6 h before measuring luciferase activity in the cell lysates. The results are the means ± SEM of three measurements and they are expressed as the percentage of inhibition, considering CD3/CD28 stimulation as 100% activation: **p≤0.01 indicates significant changes between CD3+CD28 and VCE-003 treatment.

We next assessed the effects of VCE-003 on the expression of activation markers and on the secretion of pro-inflammatory markers by CD3/CD28-stimulated primary T cells. The supernatants of these cells were analyzed using the R&D Systems Human Cytokine Array that detects the relative levels of 36 different cytokines, chemokines and acute phase proteins in a single sample (Figure S1 in File S1). We found that CD3/CD28 stimulation induced the release of: Th1 related cytokines GM-CSF, INFγ and TNFα; Th2 related cytokines, IL-17; the chemokines RANTES, MIP-1α, MIP-1β and IP-10; and the adhesion molecule sICAM-1, and pretreatment with 5 µM VCE-003 inhibited the release of all these soluble mediators (Figure 2A and 2B). The effect of VCE-003 on the cell surface expression of the activation markers CD25 (IL-2Rα) and CD54 (ICAM-1) was also studied in CD3/CD28-stimulated primary T cells. VCE-003 strongly inhibited the proportion of cells expressing the CD25 and CD54 markers at the cell surface (Figure 2C). To gain insight into the molecular mechanisms of cytokine inhibition Jurkat cells were transiently transfected with the IL-2-Luc, TNFα-Luc and IL-17-Luc plasmids that contain specific gene promoters fused to the luciferase gene. VCE-003 (5 µM) inhibited the transcriptional activity of the promoters induced by CD3/CD28 mAb stimulation by nearly 60% (p<0.01 vs. control activated cells: Figure 2D) and therefore, it is likely that the inhibitory mechanism of VCE-003 occurs both at the transcriptional and post-transcriptional levels.

### VCE-003 dampens the IL-17-induced inflammatory response of macrophages

**Figure 3:**
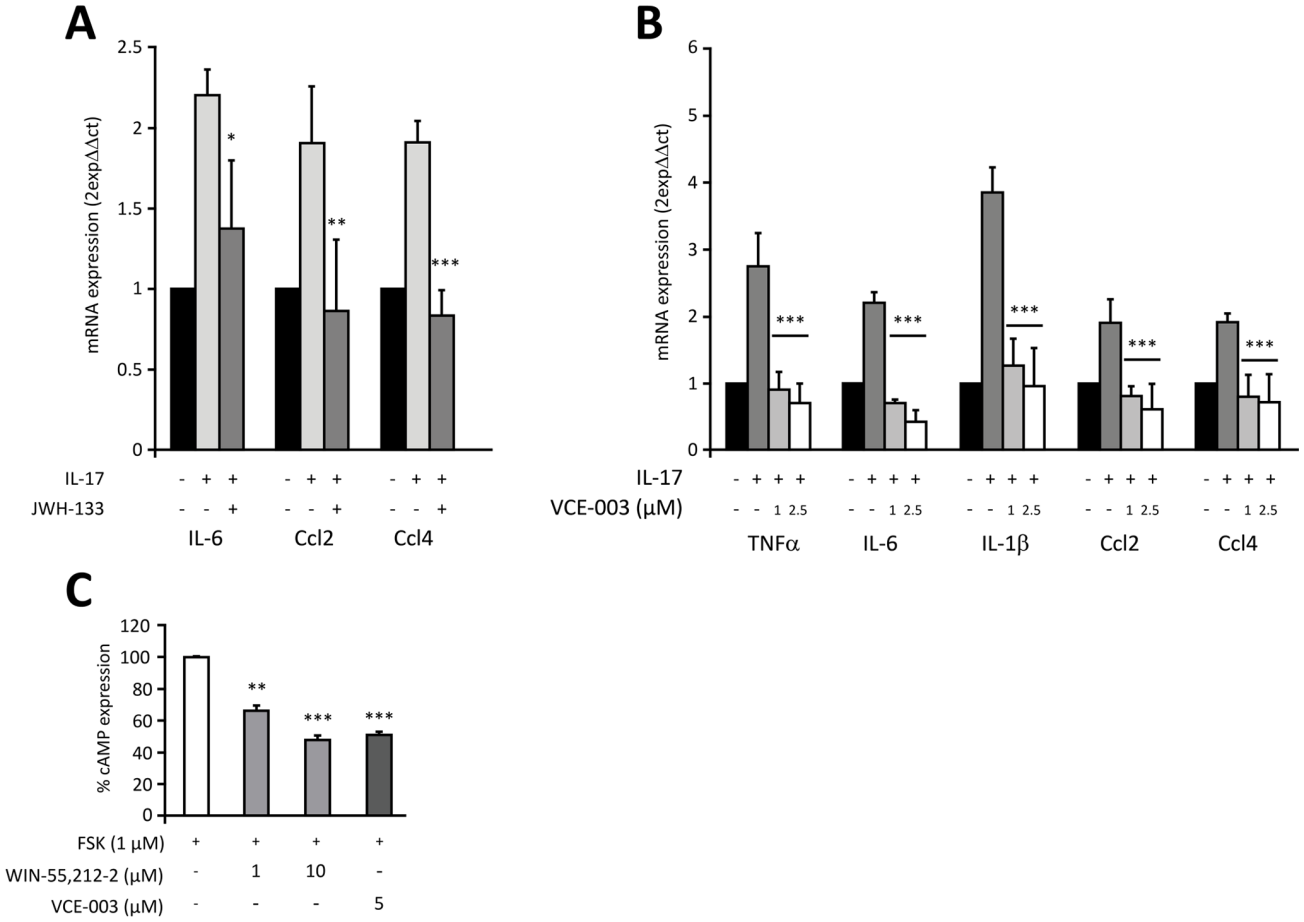
VCE-003 and JWH-133 reduces IL-17-induced M1 polarization in macrophages. Serum starved RAW264.7 cells were exposed to either JWH-133 (5 µM) or VCE-003 (1 and 2.5 µM) for 18 h and then maintained for 24 h in the presence or absence of recombinant mouse IL-17 (50 ng/ml). A) RT-PCR analysis of M1 gene expression in JWH-133-treated RAW264.7 cells. B) RT-PCR analysis of M1 gene expression in VCE-003-treated RAW264.7 cells. The results are expressed as the fold change with respect to untreated cells and they are the mean ± SEM of four independent experiments. C) CHO-CB2 cells were transiently transfected with the CRE-luc plasmid and then exposed to FSK (1 µM) for 6 h in the presence or absence of the indicated concentrations of WIN-55,212-2 (1 or 10 µM) and VCE-003 (5 µM) before luciferase activity was measured in the cell lysates. The results are expressed as the means ± SEM of three determinations and they are expressed as the percentage inhibition considering FSK stimulation as 100% activation: *p<0.05, **p<0.01 and ***p<0.005 in an unpaired two-tailed Student's *t* test.

Macrophages are also important effector cells that mediate the immune responses in EAE. They act as antigen presenting cells (APC), thereby activating an antigen-specific T cell response in the periphery and CNS. Activation of the CB2 receptor was recently shown to blunt IL-17-induction of M1 macrophages [16] and therefore, we assessed whether in addition to directly inhibiting IL-17 production by primary T cells, VCE-003 might also counteract the functional properties of IL-17 on its target cells. Treatment of RAW264.7 macrophages with IL-17 promoted their polarization towards a pro-inflammatory M1 phenotype, as shown by increased expression of M1 markers like TNFα, IL-6, IL-1β or Ccl2, and Ccl4. However, exposing RAW264.7 macrophages to either the CB2 agonist JWH-133 or VCE-003 strongly inhibited the induction of M1 markers by IL-17 (Figure 3A and 3B). Moreover, we show that VCE-003 is a functional CB2 agonist (Figure 3C) by using CHO-CB2 cells transfected with the CRE-luc plasmid and treated with FSK. Taken together, these data strongly suggest that CB2 receptor activation by VCE-003 blunts IL-17 polarization of M1 macrophages.

### VCE-003 attenuates the clinical severity and neuropathology of EAE

**Figure 4:**
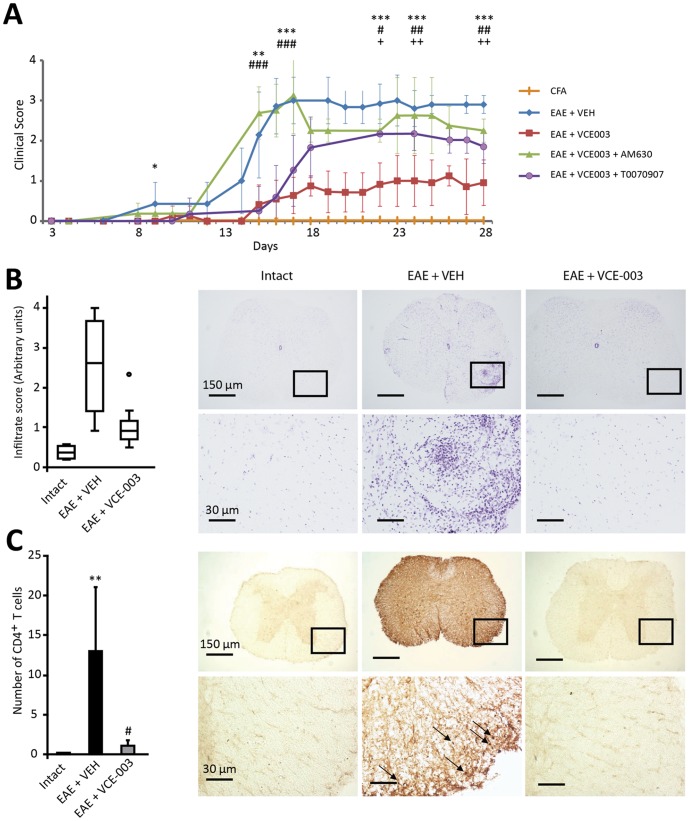
VCE-003 alleviates EAE and prevents CD4^+^ cells infiltration. (A) VCE-003 significantly ameliorates the clinical signs and disease progression of EAE (squares), and these effects are partially blocked by a CB2 antagonist (AM630, triangles) or PPARγ antagonist (T0070907, circles). The results are shown as the means ± SD: *p = 0.015 EAE + VCE-003 vs EAE + VEH; **p = 0.005 EAE + VCE-003 vs EAE + VEH; ***p<0.001 EAE + VCE-003 vs EAE + VEH; #p = 0.016 EAE + VCE-003 + AM630 vs EAE + VCE-003; ##p = 0.008 EAE + VCE-003 + AM630 vs EAE + VCE-003; ###p<0.001 EAE + VCE-003 + AM630 vs EAE + VCE-003; +p = 0,009 EAE + VCE-003 + T0070907 vs EAE + VCE-003; ++p<0.01 EAE + VCE-003 + T0070907 vs EAE + VCE-003. (B) VCE-003 reduces the number of infiltrates and (C) it significantly reduces the number of CD4^+^ T cells in thoracic spinal cord sections. The figure shows the representative staining of spinal cord sections: LFB (B) and CD4 immunohistochemistry (C). Arrows indicate CD4^+^ T cells. The results (C) are shown as the means ± SEM: **p = 0,004 vs Intact; #p = 0,030 vs EAE + VEH.

VCE-003 has been reported to display anti-inflammatory properties in the Theiler's virus model of MS [20]. On the basis of the *in vitro* results described above we investigated the therapeutic potential of VCE-003 in the autoimmune model of the disease (EAE), performing the treatments at an early stage of the disease since mice received the first injection of VCE-003 at day 6 p.i. Subcutaneous immunization with MOG_35-55_ induced EAE in all mice that received the vehicle alone, with disease kinetics in these mice consistent with previous studies given the mean onset on day 9 p.i. [23]. All vehicle-treated mice developed a severe disease that peaked by day 17 p.i., and four moribund animals were sacrificed on day 18 p.i. By contrast, the clinical manifestations of EAE were attenuated in mice receiving daily injections of VCE-003 (5 mg/kg, i.p.) from day 6 p.i. (Figure 4A). In these mice that received VCE-003, the mean onset was on day 15 p.i. and the disease peaked on day 23 p.i., not reaching a score of 1 throughout the course of the experiment (day 6-day 28).

Because VCE-003 acts as an agonist for the PPARγ receptor and it binds to the CB2 receptor [20], we wanted to determine the receptor type involved in the beneficial effects of VCE-003 in EAE. Thus, we administered a selective antagonist for the CB2 receptor (AM630) or for the PPARγ receptor (T0070907) in combination with VCE-003, and we found that that the benefits of VCE-003 were significantly counteracted by the administration of the CB2 antagonist AM630, particularly in the induction phase. In fact, the disease peaked on day 15 p.i. in both groups that received the CB2 antagonist AM630: the EAE vehicle control mice and the EAE-VCE-003 treated mice. Treatment with the PPARγ receptor antagonist, the compound T0070907, also attenuated the effects of VCE-003 but to a lesser extent, and the peak disease onset was only delayed to day 22 p.i. These data suggest the involvement of both receptors in the capacity of VCE-003 to ameliorate EAE. We also observed that EAE mice treated with VCE-003 showed significantly less infiltrates than EAE control mice that the received vehicle alone (Figure 4B). Interestingly, VCE-003 treatment reduced the number of CD4^+^ T cells in the spinal cord of EAE mice (Figure 4C).

**Figure 5:**
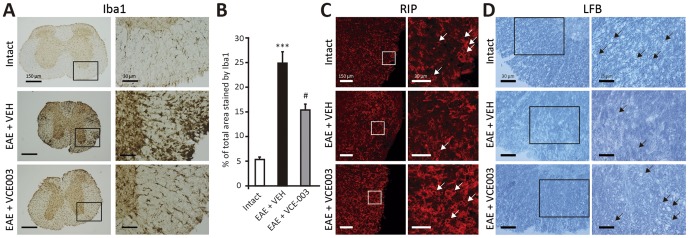
VCE-003 significantly reduces microglial activation and preserves myelin structure. (A) VCE-003 notably reduces microglial activation (Iba1^+^ cells). (B) Quantification of Iba1^+^ cells are shown as means ± SEM (***p<0.001 vs Intact; #p = 0.021 vs EAE + VEH). Thoracic spinal cord sections from symptomatic animals show a clear disruption of myelin, whereas exposure to VCE-003 contributes to maintain the myelin structure (C, RIP immunofluorescence staining; D, LFB staining).

Histopathological analysis of spinal cord tissue showed that the extensive microglia/macrophage activation in the spinal cord of EAE mice evident by Iba-1 immunohistochemistry was dramatically reduced by VCE-003 (Figure 5A) and when quantified (Figure 5B), VCE-003 significantly decreased microglial activation (p<0.05). Moreover, whereas important areas of demyelination were stained by LFB in vehicle-treated mice, a pronounced reduction in demyelination was evident in EAE mice that received VCE-003 (Figure 5D). This was confirmed by myelin protein RIP staining, which showed clear myelin disruption in the spinal cord of control EAE mice (Figure 5C) while VCE-003 administration contributed to the maintenance of myelin structure.

### VCE-003 reduces axonal damage in EAE mice

**Figure 6:**
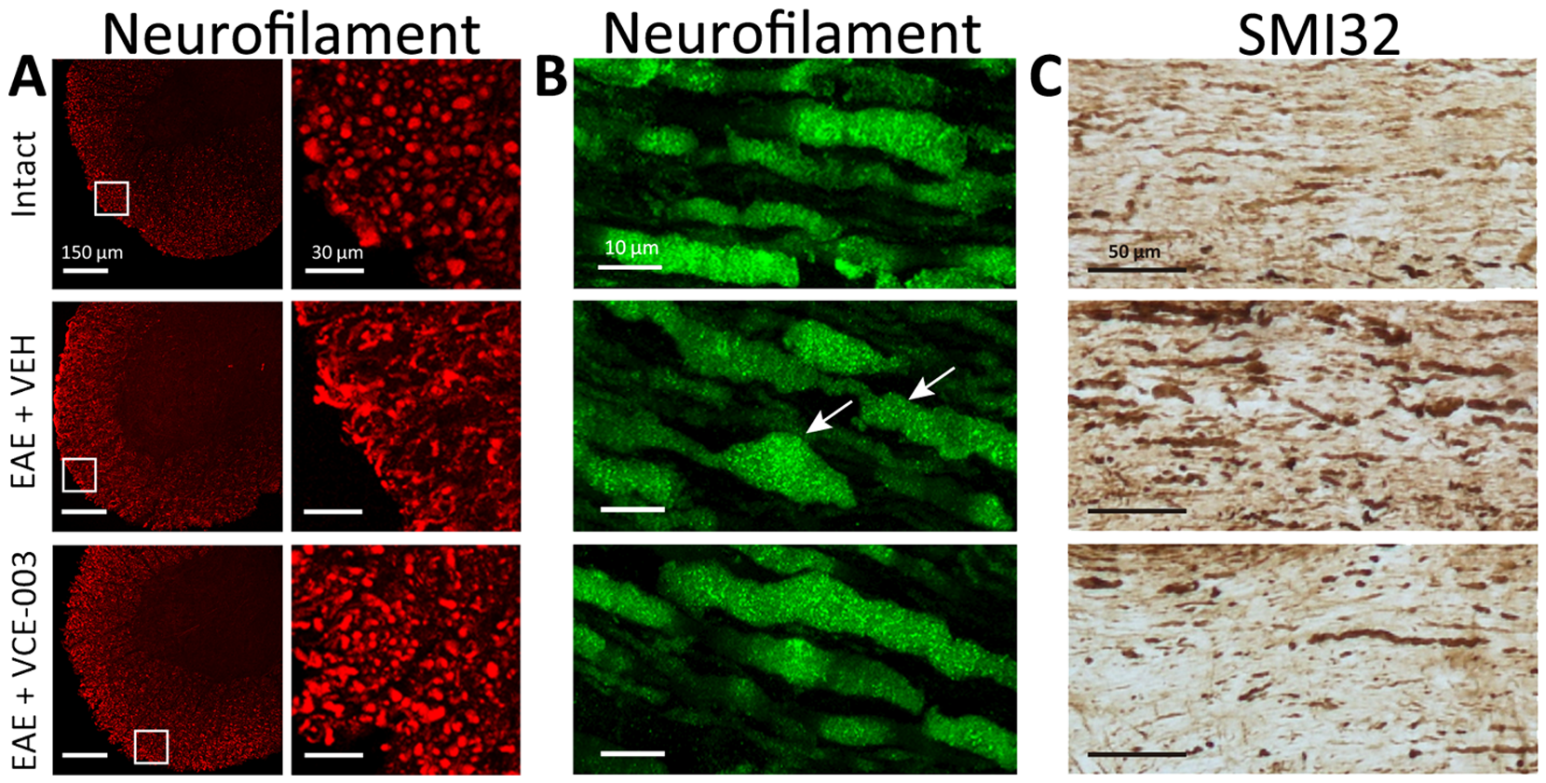
VCE-003 reduces axon damage. Representative images of axon morphology changes: (A) transversal thoracic spinal cord sections, neurofilament staining in red; (B) longitudinal thoracic spinal cord sections, neurofilament staining in green (please note axon swelling, arrows). (C) VCE-003 treatment helps to preserve the axon structure as showed in longitudinal thoracic spinal cord sections by SMI32 staining.

To further evaluate the potential of VCE-003 as neuroprotective agent, we compared the axonal damage in EAE mice that received the vehicle alone or VCE-003 by assessing neurofilament and SMI32 labeling. In representative coronal thoracic spinal cord sections, neurofilament staining highlights the disorganization of the white matter tracts in EAE control mice, in contrast to the thoracic spinal cord structure evident in intact animals or those EAE mice that received VCE-003 (Figure 6A). Longitudinal spinal cord sections stained with neurofilament (Figure 6B) show the swelling and deformation of the axons, and the ovoid formation in EAE control mice. Axon lesions were less frequent in the mice that received VCE-003 and they had markedly less damage that was particularly evident when the longitudinal spinal cord sections labeled with SMI32 (Figure 6C).

### Reduced inflammatory mediators in EAE mice that received VCE-003

**Figure 7:**
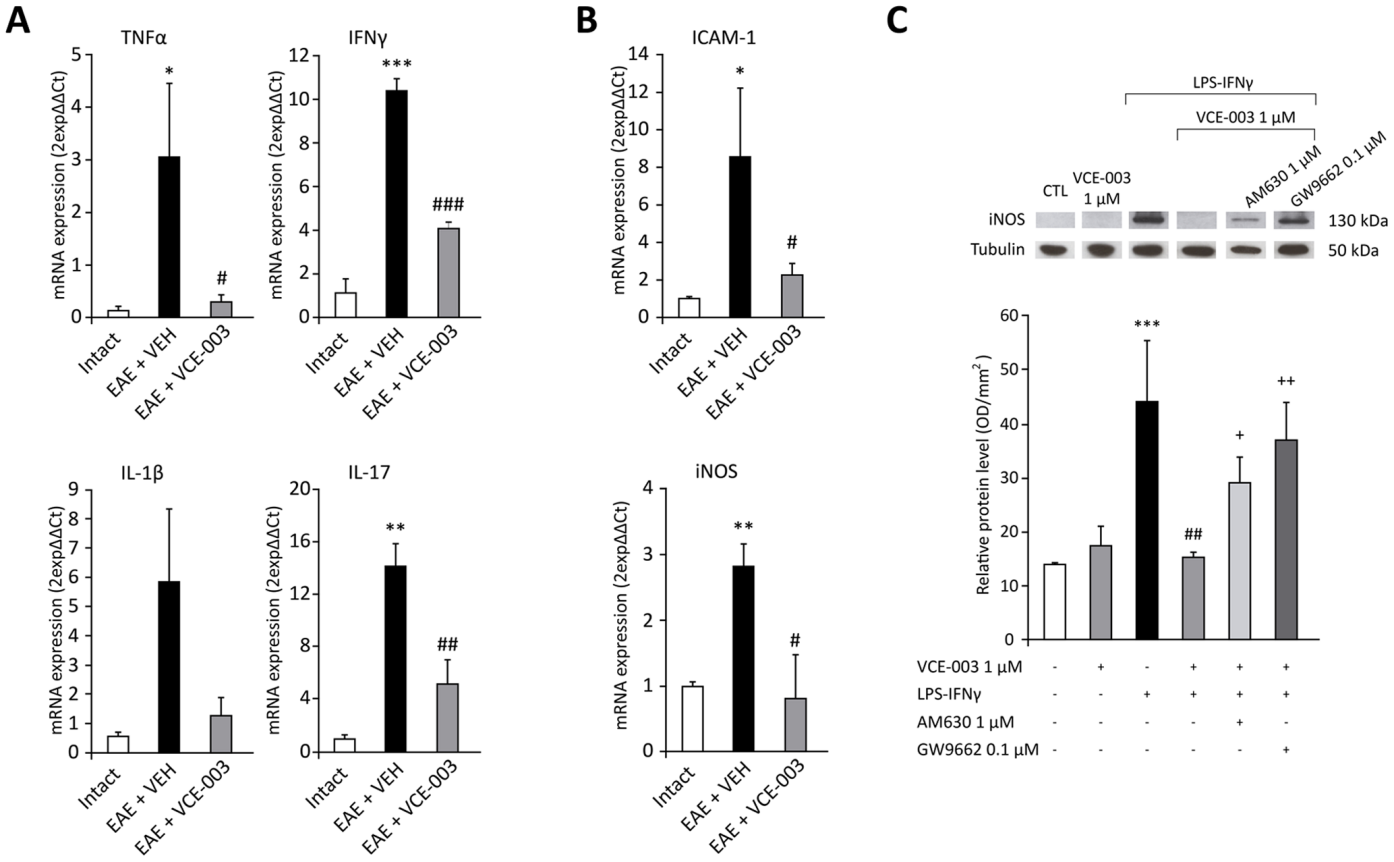
VCE-003 reduces the expression of inflammatory marker mRNAs in the spinal cord and of iNOS protein in BV2 cells. VCE-003 decreased the mRNA expression of TNFα, IFNγ, IL-1β and IL-17 (A), and the adhesion molecule ICAM-1 and iNOS (B). (C) Immunoblotting of iNOS. Proteins in lysates (30 µg) from BV2 cells stimulated with LPS (50 ng/ml) and IFNγ (100 u/ml), and pre-treated with VCE-003 (1 µM). Both CB2 and PPARγ antagonists (AM630 1 µM and GW9662 0.1 µM, respectively) were administered 10 min before than VCE-003 treatment. The results are presented as the relative ratio of proteins, where the iNOS signal obtained by densitometric analysis was normalized to the tubulin signal. VCE-003 treatment significantly reverses the LPS-IFNγ-induced effects to the control situation, and both CB2 and PPARγ antagonists blocked this effect of VCE-003. The results in (A, B) are shown as the means ± SEM: *p = 0,011 vs Intact (TNFα); *p = 0,003 vs Intact (ICAM-1); **p<0.01 vs Intact; #p = 0.05 vs EAE + VEH (ICAM-1); ##p<0.01 vs EAE + VEH; ###p<0.001 vs EAE + VEH). The results in (C) are shown as the means ± SEM from three independent experiments performed in triplicate: ***p<0.001 vs CTL; ##p<0.01 vs CTL + LPS- IFNγ; +p = 0.034 vs VCE-003 1 µM + LPS- IFNγ; ++p = 0.002 vs VCE-003 1 µM + LPS- IFNγ.

The expression of inflammatory mediators was studied by isolating total mRNA from the spinal cords of mice at chronic stages of the disease (28 days p.i.). Quantitative real time PCR was performed using primers specific for the pro-inflammatory cytokines TNFα, IFNγ, IL-1β and IL-17, and the expression of transcripts for the adhesion molecule ICAM-1 and the inducible nitric oxide synthase (iNOS) was also quantified. A similar analysis was performed with RNA isolated from the same region of the spinal cord from CFA mice. Compared with the untreated mice, there was a significant increase in all the inflammatory markers studied in EAE mice that received the vehicle alone (Figure 7A and 7B). By contrast, there was a significant decrease in TNFα, IFNγ, IL-17, ICAM-1 and iNOS expression in the EAE mice that received VCE-003, while there was also a tendency towards reduced IL-1β expression (p = 0.075). To investigate whether VCE-003 targeted microglial cells in the induction of iNOS we evaluate the expression of this protein in Western blots of cultured BV2 microglial cells (Figure 7C) in which VCE-003 (1 µM) significantly reduced the expression of iNOS induced by exposure to LPS/IFNγ. The effect of VCE-003 on iNOS expression was blocked by the CB2 receptor antagonist AM630 (1 µM) and likewise, the blockade of PPARγ receptors by GW96662 (0.1 µM) reversed the inhibitory effects of VCE-003 (1 µM) on LPS/IFNγ-induced iNOS expression. These results indicate that both CB2 and PPARγ receptors were involved in the anti-inflammatory effects of VCE-003 in BV2 cells.

## Discussion

This study provides evidence that the cannabigerol quinone derivative, VCE-003, suppresses *in vitro* T cell responses, that it dampens the pro-inflammatory cytokine production by IL-17-stimulated macrophages and attenuates the pathology development of EAE induced by MOG immunization. This improvement is evident through several measures of EAE pathology, including: a decreased neurological deficit score; reduced inflammatory cell infiltration, in particular that of CD4^+^ T cells; demyelination and axonal damage in the spinal cord.

The mechanisms underlying the improvement in EAE induced by VCE-003 treatment are not clear, although the suppression of immune and inflammatory cell activity seems to be involved. In accordance with its pharmacological profile, the activation of both PPARγ and CB2 receptors appear to be implicated in the amelioration of EAE by VCE-003 [20]. Microglia/macrophages play a dual role in the pathogenesis of MS as they contribute to lesion formation and axonal damage, but they also support repair mechanisms [24]. The activation of microglia has been closely associated with the development of histopathological lesions, and the progression of MS and EAE [5], [25]. When microglia/macrophages are activated they release some cytotoxic mediators that may provoke tissue injury, including pro-inflammatory cytokines like IL-1β, IFNγ, IL-6, TNFα, nitric oxide (NO) and ROS, [26], [27]. However, most importantly, several cytokines can reactivate microglia, which leads to feed-forward regulation of inflammation. The decrease in Iba-1 immunoreactivity following VCE-003 treatment reflected the reduced microglial activation in EAE mice. This observation suggests that VCE-003 might ameliorate EAE symptoms by dampening microglial activation in the spinal cord, thereby impairing important inflammatory steps that lead to the damage of myelin and axons. In support of this hypothesis, we found a decrease in the expression of mRNA transcripts encoding the cytokines TNFα, IFNγ and IL-1β in the spinal cord of VCE-003 EAE-treated mice, together with a marked reduction in the mRNA transcripts encoding the adhesion molecule ICAM-1 and iNOS. In addition, direct evidence that microglial cells are targets for VCE-003 [20] was confirmed when it was shown to reverse the increase in iNOS protein in activated murine BV2 microglial cells through a mechanism that involves PPARγ and CB2 receptors, supporting our findings on disease severity scores.

Myelin-specific CD4^+^ Th1 and Th17 cells [21] with the contribution of CD8^+^ T cells [28], [29], is one of the major driving forces in the pathological process of EAE. Here, we show that there were fewer CD4^+^ T cells in the spinal cord of EAE mice after VCE-003 administration and thus, the improvement in spinal cord damage may be associated with decreased CD4^+^ T cell infiltration from peripheral tissues. In this sense, we have shown that VCE-003 inhibits peripheral primary T cell activation and proliferation, as well as the release of Th1 and Th17 cytokines or chemokines. Although the lipophilic nature of VCE-003, like other pCBs, predicts it will penetrate into the CNS, it is possible that VCE-003 may also exert its effects at the level of the peripheral immune response. Accordingly, it is noteworthy that VCE-003 inhibits IL-17-mediated production of pro-inflammatory cytokines in macrophages and perhaps, the migration of pro-inflammatory macrophages to the CNS.

Preclinical and clinical data have shown that Th17 cells are associated with several autoimmune diseases, such as MS [11], arthritis, psoriasis and lupus. In the present study, IL-17 mRNA expression was reduced in the spinal cord of EAE mice administered VCE-003. *In* vitro approaches indicated that VCE-003 was capable of reducing the transcription of IL-17, and it was more effective in inhibiting TNFα release than TNFα-gene promoter activity. On this basis, VCE-003 possibly inhibits cytokine release at both the transcriptional and post-transcriptional level. Since we found that VCE-003 inhibited IL-17 signaling in macrophages we cannot rule out the possibility that a similar situation may also occur also in primary T cells. If this were the case, IL-17 released in CD3/CD28-stimulated T cells could act in an autocrine manner, increasing the stability of other CD3/CD28-induced cytokine mRNAs, an activity that could also be inhibited by VCE-003 [30], [31]. Although PPARγ and CB2 are the more relevant targets for VCE-003 we cannot discard that other mechanism(s) of action are also involved in the immunosuppressive activity of VCE-003. In this sense it is also possible that the effect of VCE-003 on cytokine expression is reflecting inhibition of T cell proliferation mediated by targeting downstream signal pathways other than PPARγ and CB2, which prevent proliferation and subsequently cytokine production. We are currently performing experiments to identify the exact mechanism of action of VCE-003 in TCR-induced and IL-17R-induced signaling pathways.

The CNS has several protective antioxidant mechanisms that are regulated through the nuclear factor–E2-related factor transcription factor (Nrf2) and the antioxidant response element (ARE). In MS, Nrf2/ARE expression is enhanced and this is indicative of a response to oxidative stress. Moreover, disruption of Nrf2 gene expression in mice exacerbates the clinical and pathological symptoms of EAE [32]. Here, we found that VCE-003 activates the Nrf2 pathway in several neuronal cell lines (Figure S2 in File S1). Nrf2 is a major influence in the upregulation of multiple antioxidant defense systems in response to oxidative stress [33]. Uncontrolled or excessive oxidative stress may increase the expression of Nrf2 mRNA, as observed in EAE mice, probably as a primary mechanism to suppress aberrant oxidative stress responses and to promote protection from inflammatory disease. However, it should be noted that VCE-003-treated EAE mice showed reduced Nrf2 transcripts and no changes in Nrf2-dependent gene expression Hmox-1 (Figure S3 in File S1). The discrepancies between the *in vitro* and *in vivo* results may be due to the fact that Nrf2 expression was assessed at the end of treatment, when the clinical scores of the mice that received VCE-003 were less than 1 and therefore, these EAE mice showed a clear clinical improvement.

BBB integrity is critical to regulate the infiltration of migrating cells into the CNS. ICAM-1 was expressed strongly in acute MS lesions and comparable levels were also detected in chronic-active MS lesions, whereas the expression of VCAM-1 was greatly increased in chronic-active MS lesions compared to in acute MS [34], [35]. In the EAE model, many studies have demonstrated changes in adhesion molecules that reflect alterations to the BBB [36]. We have recently shown that VCAM-1 is reduced in the spinal cord of TMEV-infected mice that were administered VCE-003 [20]. In keeping with the fact that VCAM-1 expression is critical to allow lymphocyte trafficking from the periphery to the CNS, we showed a decrease in spinal cord infiltrates and specifically, decreased CD4^+^ T cells. Collectively these data may explain why immunized mice that receive VCE-003 display a notable alteration in disease onset and severity, in terms of reduced symptomatology and inflammation. Remarkably, VCE-003 treated mice develop significantly less paralysis and histological signs of EAE, and concordantly, they display weaker expression of the canonical Th1 cytokine IFNγ and the Th17 cytokine, IL-17A. As VCE-003 acts as an agonist of both CB2 and PPARγ receptors, and the activation of these receptors has been linked with anti-inflammatory effects in EAE [37], [38], we showed that the blockade of these receptors significantly attenuated the anti-inflammatory effects of VCE-003 in microglia. Although more work is needed to determine the cellular and molecular targets of VCE-003, and to clearly establish the signaling pathways involved in its actions, the unique capacity of VCE-003 to simultaneously repress IL-17 expression, microglial activity and CNS infiltrates suggests that it may be useful to manage MS. This CBG derivative appears to be a novel compound for inflammatory diseases.

## Conclusions

The primary findings of this study show that VCE-003 is an immunosuppressive compound targeting PPARγ and CB2 receptors. VCE-003 inhibits Th1 and Th17 responses and IL-17-induced polarization of M1 macrophages. Treatment with VCE-003 attenuated EAE in mice and we observed a reduction of CD4^+^ cells infiltrates in the spinal cord accompanied by decreased microglial activation, myelin sheets structure preservation and reduced axonal damage. At the concentration tested VCE-003 does not show cytotoxicity in primary T cells and represents a potential therapeutic agent for the treatment of human diseases with both inflammatory and autoimmune components.

## Supporting Information

File S1 **Figures S1–S3.** Figure S1. Effects of VCE-003 on cytokines and chemokines in T cells. Human peripheral T cells were stimulated for 72 h with the OKT3 (1 µg/ml) and anti-CD28 (0.5 µg/ml) mAbs in the presence or absence of increasing concentrations of VCE-003, and the culture supernatants were collected and assayed for cytokines and chemokines. Original X-ray films from the detection of cytokines and chemokines using the semiquantitative Human Cytokine Array Kit, Panel A (R&D System; Minneapolis, MN, USA). Figure S2. VCE-003 effects in Nrf2 transcription in different cell lines. The cells were transfected with the ARE-Luc plasmid and then stimulated with either CBG or VCE-003 for 6 h. The luciferase activity was measured and results are presented as the fold induction over untreated cells. The results are expressed as the means ± SEM of three determinations in triplicate. Statistical analysis: *p<0.05; **p<0.01; ***p<0.001 vs controls. Figure S3. VCE-003 effects in Nrf2 and Hmox-1 mRNA expression in spinal cord of EAE mice. Levels of mRNA expression for Nrf2 and Hmox-1 in EAE mice that received vehicle and EAE mice treated with VCE-003. *p<0.05 vs Intact; #p<0.05 vs EAE + vehicle.
(ZIP)
