# Supplementary figures and images for: A Cannabigerol Derivative Suppresses Immune Responses and Protects Mice from Experimental Autoimmune Encephalomyelitis
Source: PLoS One. 2014 Apr 11;9(4):e94733. doi: 10.1371/journal.pone.0094733 (PMC3984273; doi:10.1371/journal.pone.0094733)

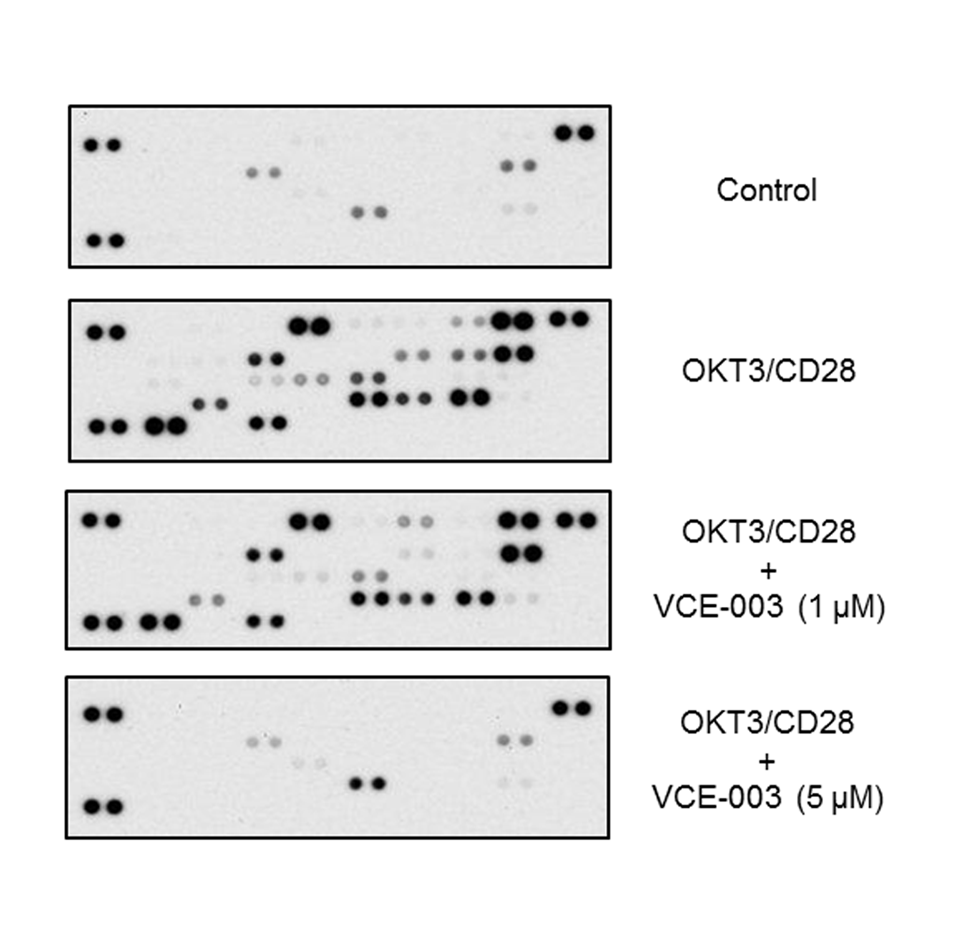

Supplement: File S1 — Figures S1–S3. Figure S1. Effects of VCE-003 on cytokines and chemokines in T cells. Human peripheral T cells were stimulated for 72 h with the OKT3 (1 µg/ml) and anti-CD28 (0.5 µg/ml) mAbs in the presence or absence of increasing concentrations of VCE-003, and the culture supernatants were collected and assayed for cytokines and chemokines. Original X-ray films from the detection of cytokines and chemokines using the semiquantitative Human Cytokine Array Kit, Panel A (R&D System; Minneapolis, MN, USA). Figure S2. VCE-003 effects in Nrf2 transcription in different cell lines. The cells were transfected with the ARE-Luc plasmid and then stimulated with either CBG or VCE-003 for 6 h. The luciferase activity was measured and results are presented as the fold induction over untreated cells. The results are expressed as the means ± SEM of three determinations in triplicate. Statistical analysis: *p<0.05; **p<0.01; ***p<0.001 vs controls. Figure S3. VCE-003 effects in Nrf2 and Hmox-1 mRNA expression in spinal cord of EAE mice. Levels of mRNA expression for Nrf2 and Hmox-1 in EAE mice that received vehicle and EAE mice treated with VCE-003. *p<0.05 vs Intact; #p<0.05 vs EAE + vehicle. (ZIP) [file pone.0094733.s001.zip › S1.tif]

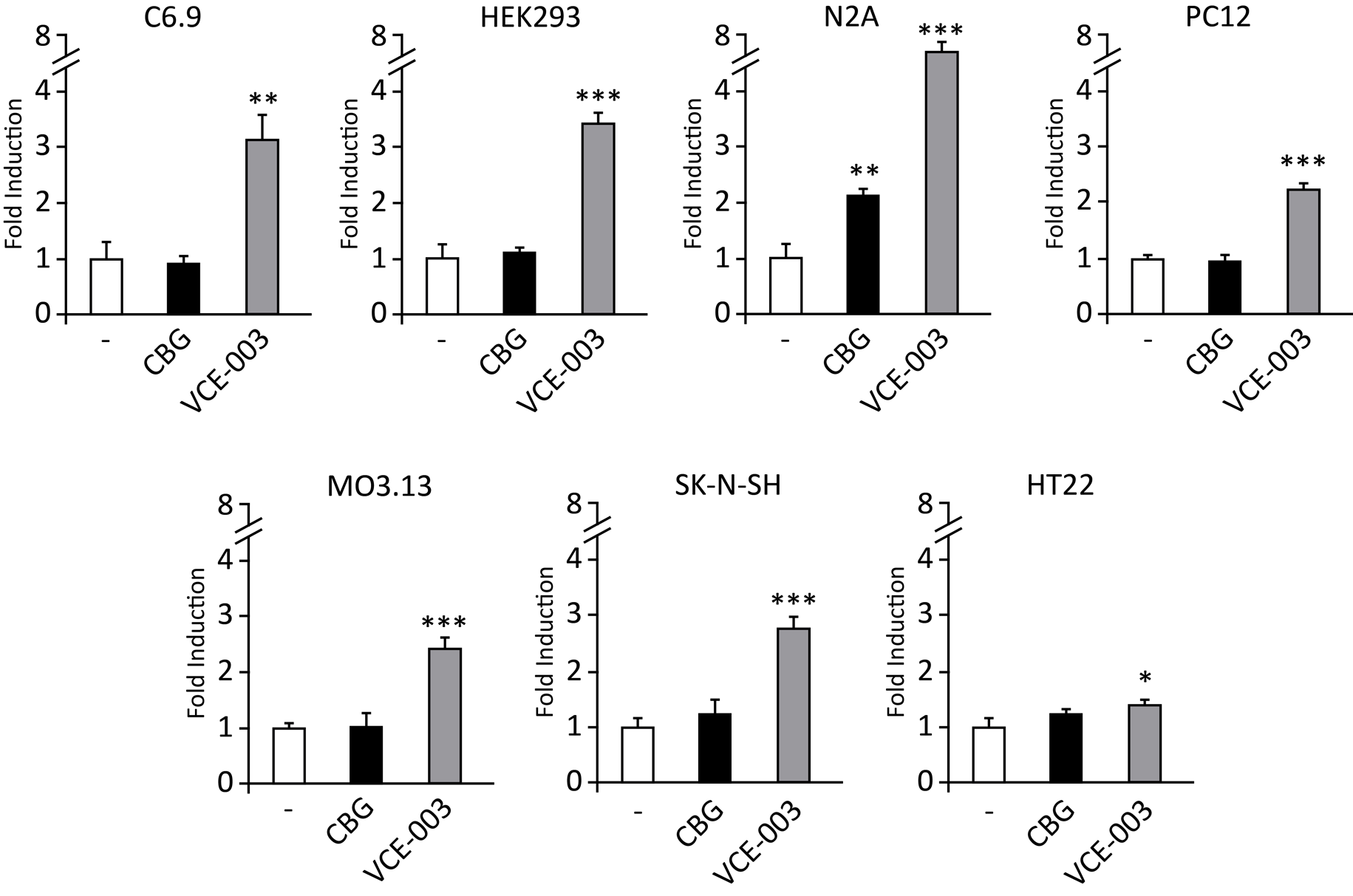

Supplement: File S1 — Figures S1–S3. Figure S1. Effects of VCE-003 on cytokines and chemokines in T cells. Human peripheral T cells were stimulated for 72 h with the OKT3 (1 µg/ml) and anti-CD28 (0.5 µg/ml) mAbs in the presence or absence of increasing concentrations of VCE-003, and the culture supernatants were collected and assayed for cytokines and chemokines. Original X-ray films from the detection of cytokines and chemokines using the semiquantitative Human Cytokine Array Kit, Panel A (R&D System; Minneapolis, MN, USA). Figure S2. VCE-003 effects in Nrf2 transcription in different cell lines. The cells were transfected with the ARE-Luc plasmid and then stimulated with either CBG or VCE-003 for 6 h. The luciferase activity was measured and results are presented as the fold induction over untreated cells. The results are expressed as the means ± SEM of three determinations in triplicate. Statistical analysis: *p<0.05; **p<0.01; ***p<0.001 vs controls. Figure S3. VCE-003 effects in Nrf2 and Hmox-1 mRNA expression in spinal cord of EAE mice. Levels of mRNA expression for Nrf2 and Hmox-1 in EAE mice that received vehicle and EAE mice treated with VCE-003. *p<0.05 vs Intact; #p<0.05 vs EAE + vehicle. (ZIP) [file pone.0094733.s001.zip › S2.tif]

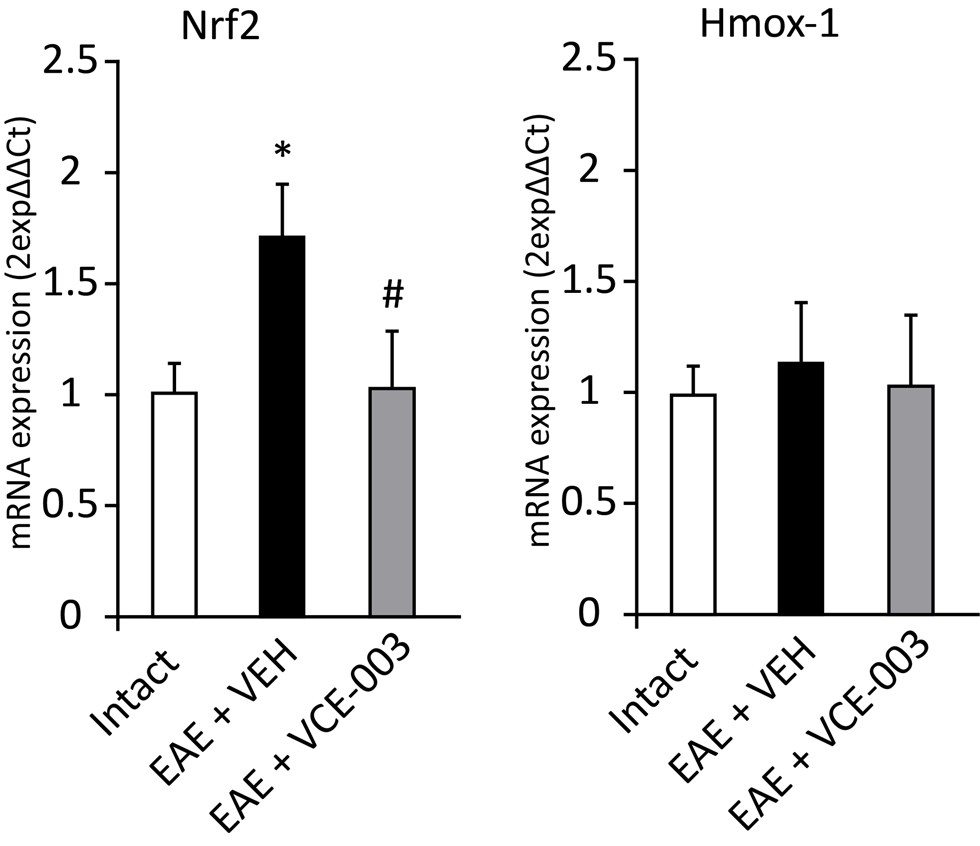

Supplement: File S1 — Figures S1–S3. Figure S1. Effects of VCE-003 on cytokines and chemokines in T cells. Human peripheral T cells were stimulated for 72 h with the OKT3 (1 µg/ml) and anti-CD28 (0.5 µg/ml) mAbs in the presence or absence of increasing concentrations of VCE-003, and the culture supernatants were collected and assayed for cytokines and chemokines. Original X-ray films from the detection of cytokines and chemokines using the semiquantitative Human Cytokine Array Kit, Panel A (R&D System; Minneapolis, MN, USA). Figure S2. VCE-003 effects in Nrf2 transcription in different cell lines. The cells were transfected with the ARE-Luc plasmid and then stimulated with either CBG or VCE-003 for 6 h. The luciferase activity was measured and results are presented as the fold induction over untreated cells. The results are expressed as the means ± SEM of three determinations in triplicate. Statistical analysis: *p<0.05; **p<0.01; ***p<0.001 vs controls. Figure S3. VCE-003 effects in Nrf2 and Hmox-1 mRNA expression in spinal cord of EAE mice. Levels of mRNA expression for Nrf2 and Hmox-1 in EAE mice that received vehicle and EAE mice treated with VCE-003. *p<0.05 vs Intact; #p<0.05 vs EAE + vehicle. (ZIP) [file pone.0094733.s001.zip › S3.tif]
