# Supplementary material for: A Cannabigerol Derivative Suppresses Immune Responses and Protects Mice from Experimental Autoimmune Encephalomyelitis
Source: PLoS One. 2014 Apr 11;9(4):e94733. doi: 10.1371/journal.pone.0094733 (PMC3984273; doi:10.1371/journal.pone.0094733)
Supplement: File S1 — Figures S1–S3. Figure S1. Effects of VCE-003 on cytokines and chemokines in T cells. Human peripheral T cells were stimulated for 72 h with the OKT3 (1 µg/ml) and anti-CD28 (0.5 µg/ml) mAbs in the presence or absence of increasing concentrations of VCE-003, and the culture supernatants were collected and assayed for cytokines and chemokines. Original X-ray films from the detection of cytokines and chemokines using the semiquantitative Human Cytokine Array Kit, Panel A (R&D System; Minneapolis, MN, USA). Figure S2. VCE-003 effects in Nrf2 transcription in different cell lines. The cells were transfected with the ARE-Luc plasmid and then stimulated with either CBG or VCE-003 for 6 h. The luciferase activity was measured and results are presented as the fold induction over untreated cells. The results are expressed as the means ± SEM of three determinations in triplicate. Statistical analysis: *p<0.05; **p<0.01; ***p<0.001 vs controls. Figure S3. VCE-003 effects in Nrf2 and Hmox-1 mRNA expression in spinal cord of EAE mice. Levels of mRNA expression for Nrf2 and Hmox-1 in EAE mice that received vehicle and EAE mice treated with VCE-003. *p<0.05 vs Intact; #p<0.05 vs EAE + vehicle. (ZIP) [file pone.0094733.s001.zip › Supplementary information.doc]

**SUPPLEMENTARY INFORMATION**

**A CANNABIGEROL DERIVATIVE SUPPRESSES IMMUNE RESPONSES AND PROTECTS MICE FROM EXPERIMENTAL AUTOIMMUNE ENCEPHALOMYELITIS**

Francisco J. Carrillo-Salinas^1^, Carmen Navarrete^2^, Miriam Mecha^1^, Ana Feliú^1^, Juan A. Collado^3^, Irene Cantarero^3^, María L. Bellido^2^, Eduardo Muñoz^3*^ and Carmen Guaza^1*^.

^1^Neuroimmunology Group, Functional and System Neurobiology Department, Instituto Cajal. Madrid, Spain. ^2^VivaCell Biotechnology España, Parque Científico Tecnológico de Córdoba. Córdoba, Spain. ^3^Instituto Maimónides de Investigación Biomédica de Córdoba (IMIBIC), Hospital Universitario Reina Sofía, Universidad de Córdoba. Córdoba, Spain.

**METHODS**

**Transient transfections and Luciferase assays*.*** HEK293, SK-N-SH, MO3.13, HT22. PC12, C6.9 and N2A cells (10^5^/ml) were transiently transfected with the plasmid ARE-Luc. The transfections were performed using Lipofectine™ reagent (Life Technologies; Madrid, Spain) according to the manufacturer’s recommendations. Twenty-four hours after transfection, the cells were treated with either CBG or VCE-003 at 10 μM for 6 h. Luciferase activity was measured as described and the specific transactivation expressed as a fold induction over untreated cells.

**Real-time RT-PCR**. Total RNA was isolated from spinal cord tissue from controls (CFA), EAE + VEH and EAE + VCE-003 treated animals. Spinal cord sections from 3 animals per group were analyzed. One μg of RNA was retrotranscribed using the iScript^TM^ cDNA Synthesis Kit and the cDNA was analyzed by real-time PCR, using the iQ^TM^ SYBR Green Supermix^.^ Detection of Hmox-1 gene expressión was detetced by qRT-PCR using a CFX96 Real-Time PCR Detection System. The GAPDH housekeeping gene was used to standardize the mRNA expression levels in every sample. Expression levels were calculated using the 2^-ΔΔCt^ method.
